# Temporal Fat Pad Filler Injection for Lifting Purposes: Shifting of the Superficial Layer of Deep Temporal Fascia

**DOI:** 10.1111/jocd.70023

**Published:** 2025-02-25

**Authors:** Kyu‐Ho Yi, Jovian Wan, Soo‐Bin Kim, Hee‐Jin Kim

**Affiliations:** ^1^ Division in Anatomy and Developmental Biology, Department of Oral Biology, Human Identification Research Institute, BK21 FOUR Project Yonsei University College of Dentistry Seoul Korea; ^2^ Maylin Clinic (Apgujeong) Seoul Korea; ^3^ Asia‐Pacific Aesthetic Academy Hong Kong China; ^4^ Department of Oral Anatomy, Institute of Biomaterial Implant, College of Dentistry Wonkwang University Iksan South Korea

**Keywords:** deep temporal fascia, dermal filler, filler, polylactic acid, temporal fat pad

## 1 Introduction

The temporal region is a critical yet often overlooked area in facial aesthetics, contributing to the harmony and balance of the upper face [1, 2]. However, as we age, this region undergoes significant anatomical changes that lead to a hollowed, concave appearance, contributing to an overall aged and tired look. The temporal hollowing often becomes a primary concern for individuals seeking facial rejuvenation, as it disrupts the smooth contours that are characteristic of a youthful appearance [3, 4, 5].

The restoration of volume in the temporal region through the use of dermal fillers has gained popularity as an effective, non‐surgical approach to address these age‐related changes. However, achieving optimal results requires a comprehensive understanding of the complex anatomy of the temporal region and the dynamic processes involved in aging [1, 2, 5, 6]. This clinical commentary will delve into the anatomical considerations crucial for successful temporal filler injections and discuss the implications of these insights for enhancing the lifting effect and achieving a natural, rejuvenated appearance.

## 2 Anatomical Considerations

The anatomy of the temporal region is intricate, with 13 distinct layers contributing to both its function and aesthetic appearance. These layers include the epidermis, dermis, subcutaneous tissue, superficial temporal fascia, innominate fascia, superficial layer of deep temporal fascia (DTF), temporal fat pad, deep layer of DTF, temporal extension of the buccal fat pad, superficial layer of the temporalis muscle, tendinous portion of temporalis, deep layer of the temporalis muscle, and the underlying bony framework. Together, these structures interact dynamically to maintain facial structure and enable movement [1, 4, 7].

## 3 Superficial and Deep Fat Compartments

The temporal region contains several distinct fat compartments broadly classified into superficial and deep layers. The superficial compartments, including the subcutaneous fat compartments located just above the superficial temporal fascia, play a vital role in maintaining a smooth, convex contour of the temples by providing volume and support to the overlying skin—contributing to the fullness and youthful appearance of the upper face. In contrast, the deep compartments lie between two layers of the DTF, covering the temporalis muscle and encompassing structures such as the temporal fat pad and the temporal extension of the buccal fat pad above the temporalis muscle. These deeper layers help stabilize the underlying skeletal and muscular framework, preserving overall facial balance.

As the aging process progresses, the temporal fat compartments—which include both superficial and deep layers—tend to thin in their upper and middle regions while thickening in the lower portion, creating a hollowed, scalloped appearance in the temples. Further atrophic changes across these compartments reduce overall volume, emphasizing the hollowing effect. Recognizing and delineating these layers, as well as understanding their specific aging patterns, is therefore essential for precise treatment planning and optimal outcomes.

## 4 Deep Temporal Fascia (DTF)

The DTF is a dense, aponeurotic layer that covers the temporalis muscle and extends downward over the lateral side of the head. The DTF is divided into superficial and deep layers, each serving a specific function in the structural integrity of the temporal region. The superficial layer of the DTF, in particular, is of significant importance in aesthetic procedures [7, 8].

This superficial layer of the DTF is a dynamic plane that moves pulls upward in response to changes in the underlying structures, such as the augmentation of the temporal fat pad. When the temporal fat pad is augmented with filler, the superficial layer of the DTF is pulled upward, contributing to the lifting of the overlying tissues. This movement is a critical mechanism in achieving a natural and effective lifting effect during filler injections.

The DTF also plays a crucial role in anchoring the overlying soft tissues to the underlying bone. As the bone remodels and the soft tissues atrophy with age, the support provided by the DTF becomes increasingly important in maintaining the structural integrity of the face. Understanding the anatomy and function of the DTF is essential for optimizing filler injection techniques and achieving a successful aesthetic outcome.

The dynamic movement of the superficial layer of DTF during filler augmentation is illustrated in Video S1, S2 demonstrating the ultrasound‐guided temporal fat pad filler injection for facial lifting using Juvelook Volume. This video highlights the key anatomical landmarks and shows how the superficial layer of DTF shifts upward, enhancing the lifting effect of the filler [4, 9].

## 5 Clinical Case

A 55‐year‐old woman presented with concerns about temporal hollowing, which contributed to a tired and aged appearance. To address this, PDLLA hybrid HA filler was chosen for its dual benefits of immediate volume restoration and long‐term collagen stimulation.

The treatment involved injecting 0.5 mL of the filler into each temple using a needle with a bolus technique under ultrasound guidance. The filler was carefully placed in temporal fat pad, it provided structural support by addressing the primary volume loss. The lifting effect was evaluated through ultrasound observation and visual analysis, allowing for precise assessment of the changes. The patient was treated with five sessions at intervals of 1 month. Post‐treatment photographs were taken 3 weeks after the final session to ensure the results.

**FIGURE 1:**
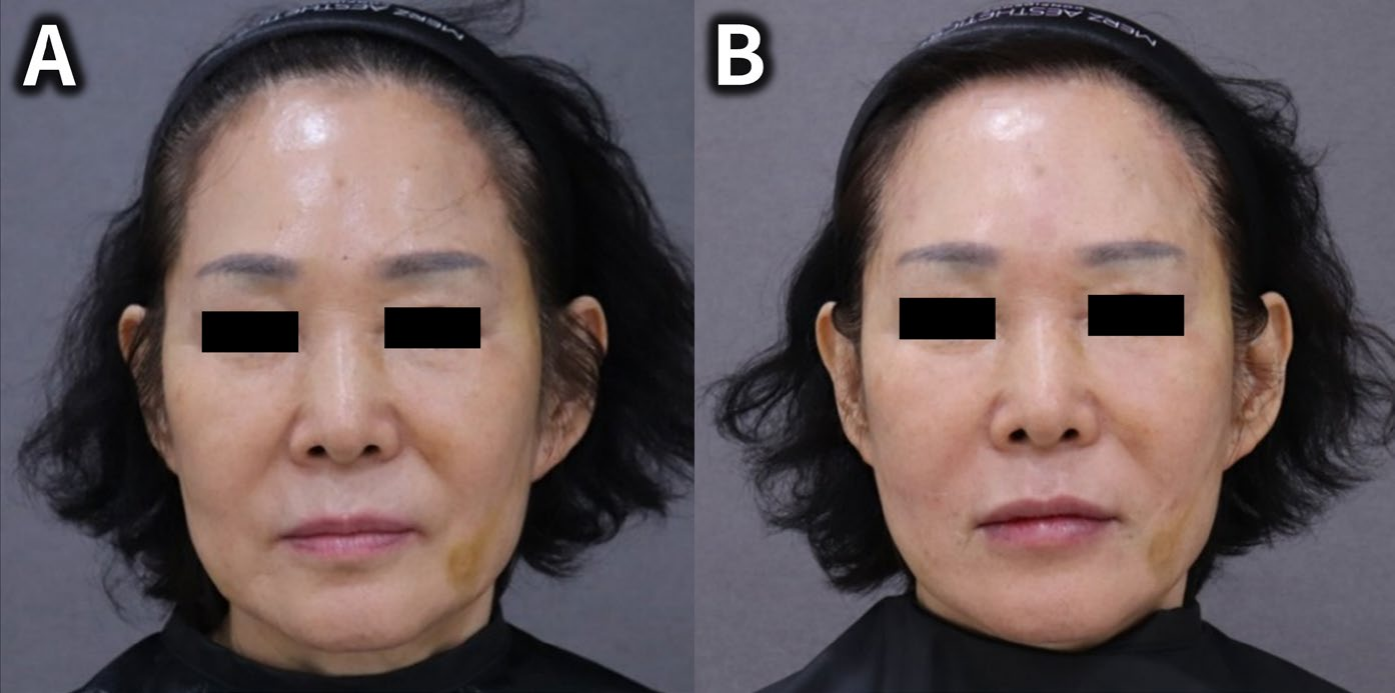
Before treatment (A): The patient exhibits significant hollowing and a concave appearance in the temporal region, contributing to an aged look. After treatment (B): The patient displays restored volume and improved facial contour in the temporal region following the injection of poly‐D,L‐lactic acid hybrid hyaluronic acid filler.

**FIGURE 2:**
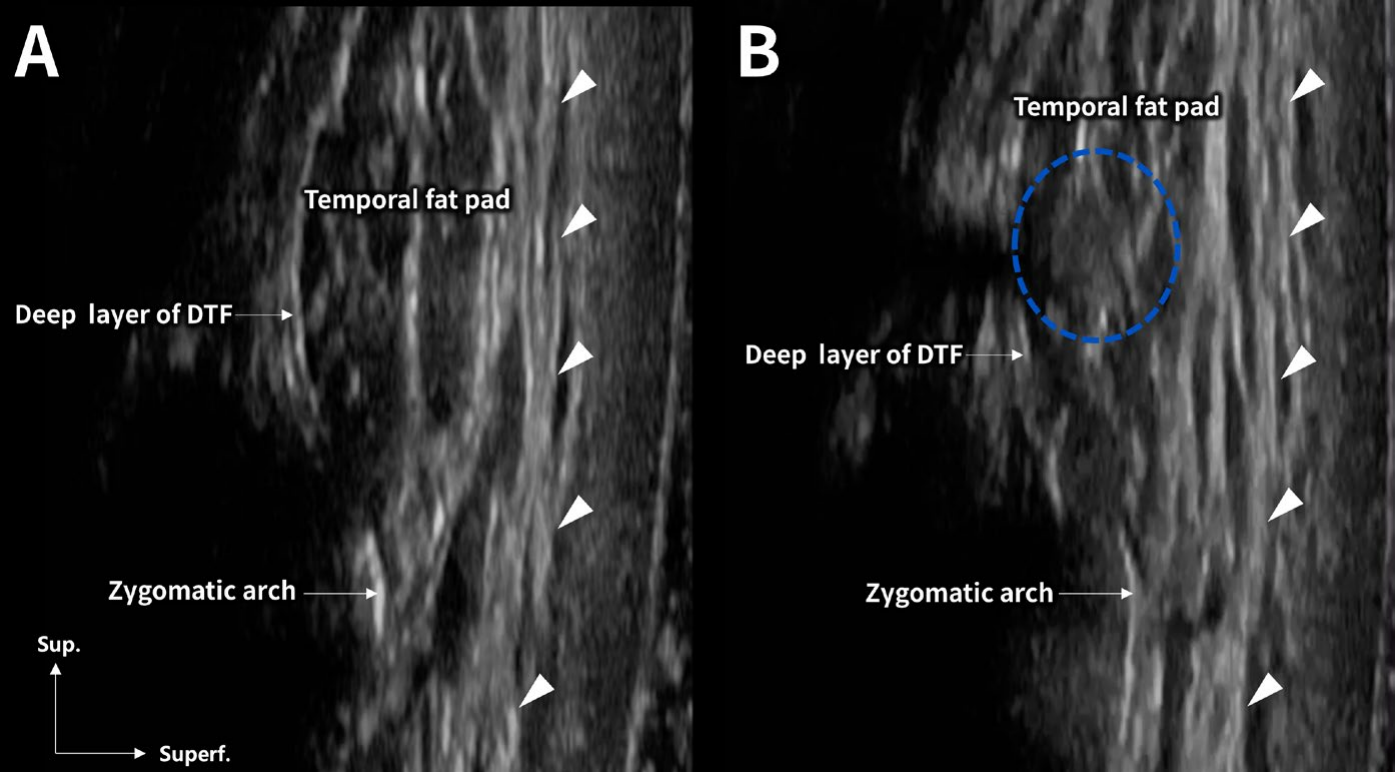
Before injection (A): Ultrasound image showing the superficial layer of the deep temporal fascia (DTF) prior to filler injection. After injection (B): Ultrasound image of the superficial layer of the DTF, demonstrating an upward shift and enhanced tension following filler injection, with the blue circular marker indicating the placement of poly‐D,L‐lactic acid hybrid hyaluronic acid filler (Juvelook Volume). The white arrowhead points to the superficial layer of the DTF.

Figures 1 and 2 depict the patient's appearance and procedural outcomes before and after treatment. Figure 1A highlights significant temporal hollowing before the procedure, while Figure 1B illustrates restored volume and improved facial contour after the injection. Figure 2 presents ultrasound images of the superficial layer of the DTF before and after the filler injection (Figure 2A,B). The post‐injection image demonstrates an upward shift and enhanced tension in the superficial layer, supporting the observed lifting effect (Figure 2B).

During the ultrasound‐guided procedure, precise filler placement minimized the risk of injury to neurovascular structures, and the dynamic movement of the superficial DTF layer was observed as the temporal fat pad was augmented, further contributing to the lifting effect.

## 6 Discussion

Restoring volume in the temporal region through dermal filler injections is a sophisticated procedure that requires a deep understanding of the underlying anatomy and the changes that occur with aging. The goal of these injections is not merely to fill the hollowed areas but to achieve a lifting effect that rejuvenates the face and restores a youthful contour [7].

The choice of PDLLA hybrid HA filler in this case was influenced by its unique properties, which make it particularly effective for temporal filler injections. This filler combines the benefits of immediate volumization from HA with the long‐term collagen‐stimulating effects of PDLLA [1, 8, 10, 11]. The PDLLA component promotes collagen synthesis, providing a more durable and natural‐looking result over time, while the HA ensures immediate improvement in volume and hydration [12, 13, 14]. This combination is ideal for the temporal region, where both volume loss and skin laxity are concerns.

**FIGURE 3:**
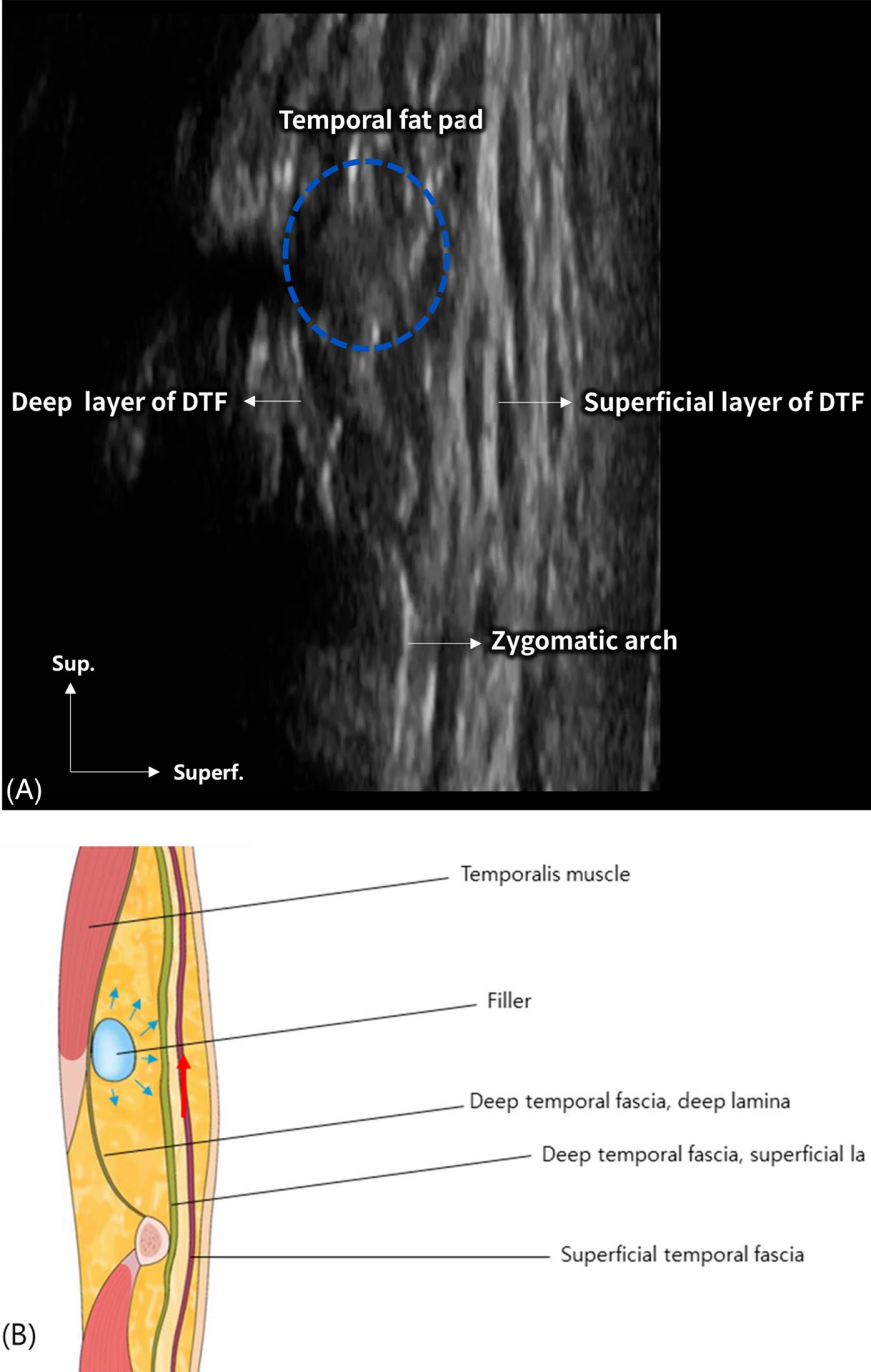
Ultrasound image illustrating the dissection of the temple in a longitudinal plane (A). The image clearly shows the placement of poly‐D,L‐lactic acid hybrid hyaluronic acid filler (Juvelook Volume) within the temporal fat pad, indicated by the blue circular marker (B). The relationship between the filler, the superficial layer of the deep temporal fascia (DTF), and surrounding structures is highlighted, emphasizing the importance of anatomical precision in achieving successful outcomes.

The relationship between the filler placement and the surrounding anatomical structures is demonstrated in Figure 3, an ultrasound image illustrating the dissection of the temple in a longitudinal plane. The image clearly shows the placement of filler within the temporal fat pad, highlighted by a blue circle. The figure emphasizes the importance of precise filler placement in achieving the desired aesthetic outcome, particularly in relation to the superficial layer of the DTF.

The temporal region also presents certain risks due to the concentration of neurovascular structures in the area. The anterior half of the lower temporal compartment is identified as a “zone of caution” because it contains critical structures such as the sentinel vein and the temporal branches of the facial nerve. Injury to these structures can result in significant complications, including nerve damage and vascular injury, making it essential for practitioners to exercise caution when injecting in this area [4, 7, 14].

The choice of cannula entry site is another important consideration in temporal filler injections. For safe and effective injections, the recommended entry point is at the medial side of the intersection of the hairline and temporal line. This site minimizes the risk of injuring critical structures and allows for precise placement of the filler in both the superficial and deep compartments. By selecting an appropriate entry point, practitioners can ensure that the filler is delivered accurately and safely, reducing the risk of complications.

The dynamic nature of the superficial layer of the DTF, particularly its upward movement during filler augmentation, underscores the importance of precise technique in these procedures [7]. The upward shift of the superficial layer of DTF, often represented as the “green line” in imaging studies, is a visual confirmation of the lifting effect that can be achieved with filler augmentation. This anatomical feature is central to the success of temporal filler injections and should be a focal point in clinical practice.

According to Byun et al. [15], PDLLA is known to stimulate preadipocytes and contribute to volume restoration. The study demonstrated that poly‐D,L‐lactic acid (PDLLA) fillers enhance adipogenesis by promoting M2 macrophage polarization and increasing fibroblast growth factor 2 (FGF2) levels, which improve the survival and proliferation of adipose‐derived stem cells (ASCs). These effects result in increased subcutaneous adipose tissue thickness in both an in vitro senescence model and aged animal skin. This may explain why the temporal fat pad exhibited increased volume, in addition to its own volumizing effect through collagen restoration.

Despite the promising outcomes observed in this study, several limitations should be acknowledged. First, the sample size is limited to a single clinical case, which restricts the generalizability of the findings. Further studies with larger sample sizes are necessary to confirm the efficacy and safety of the injection techniques described. Second, while the study demonstrates the lifting effect through ultrasound imaging and photographic comparisons, objective quantitative measurements such as three‐dimensional volumetric analysis or biomechanical assessments were not included. This may limit the ability to comprehensively evaluate the effectiveness of the procedure. Third, the long‐term outcomes of using PDLLA hybrid hyaluronic acid filler in the temporal region were not explored. Future studies should assess the durability of the lifting effect and the potential for complications over extended follow‐up periods.

In conclusion, the temporal region is a vital area for facial rejuvenation, and effective treatment requires a detailed understanding of its anatomy and the aging process. By incorporating these insights into filler injection techniques, practitioners can optimize results, restore youthful contours, and improve patient satisfaction. Careful consideration of neurovascular structures and precise placement of filler in relation to the superficial layer of the DTF are critical factors in achieving a successful aesthetic outcome.

## Author Contributions

**Conceptualization:** Kyu‐Ho Yi; Soo‐Bin Kim; Jovian Wan. **Writing – Original Draft Preparation:** Kyu‐Ho Yi; Soo‐Bin Kim; **Writing – Review and Editing:** Kyu‐Ho Yi; Soo‐Bin Kim; **Visualization:** Jovian Wan. **Supervision:** Hee‐Jin Kim. All authors have reviewed and approved the article for submission.

## Conflicts of Interest

The authors declared no potential conflicts of interest with respect to the research, authorship, and publication of this article. This study was conducted in compliance with the principles set forth in the Declaration of Helsinki.

## Supporting information


Video S1.

Video S2.

## Data Availability

The data that support the findings of this study are available from the corresponding author upon reasonable request.

## References

[1] S. B. Kim , H. Hu , H. Bae , and K. H. Yi , “Anatomy of the Temporal Region to Guide Filler Injections,” Surgical and Radiologic Anatomy 46, no. 5 (2024): 615–624. 38480594 10.1007/s00276-024-03340-x

[2] W. Lee , J. W. Park , and E. J. Yang , “Temple Augmentation by Injecting a Hyaluronic Acid Filler Between the Superficial and Deep Temporal Fasciae,” Journal of Cosmetic Dermatology 21, no. 10 (2022): 4313–4318. 35435310 10.1111/jocd.15004

[3] A. Swift , S. Liew , S. Weinkle , J. K. Garcia , and M. B. Silberberg , “The Facial Aging Process From the “Inside out”,” Aesthetic Surgery Journal 41, no. 10 (2021): 1107–1119. 33325497 10.1093/asj/sjaa339 PMC8438644

[4] R. L. Huang , Y. Xie , W. Wang , et al., “Anatomical Study of Temporal Fat Compartments and Its Clinical Application for Temporal Fat Grafting,” Aesthetic Surgery Journal 37, no. 8 (2017): 855–862. 28520850 10.1093/asj/sjw257 PMC5846703

[5] C. S. Yang , Y. L. Huang , C. B. Chen , et al., “Aging Process of Lateral Facial Fat Compartments: A Retrospective Study,” Aesthetic Surgery Journal 41, no. 6 (2021): 247–254. 10.1093/asj/sjaa340 33649752

[6] J. E. Cohn , T. Pion , and T. M. Greco , “"Not Above, Not Below: Right in the Middle!"‐Novel Filler Technique for Temporal Augmentation and Rejuvenation,” Facial Plastic Surgery 36, no. 5 (2020): 623–627. 32443157 10.1055/s-0040-1709710

[7] H. J. Lee , H. M. Kim , H. S. Ahn , J. H. Lee , and H. J. Kim , “Novel Clinical Anatomical Consideration of the Superficial and Deep Layers of the Deep Temporal Fascia,” Plastic and Reconstructive Surgery 153, no. 3 (2024): 591–599. 37010473 10.1097/PRS.0000000000010507

[8] E. E. Beheiry and F. A. M. Abdel‐Hamid , “An Anatomical Study of the Temporal Fascia and Related Temporal Pads of Fat,” Plastic and Reconstructive Surgery 119, no. 1 (2007): 136–144. 17255667 10.1097/01.prs.0000245068.04942.a8

[9] C. Bohr , J. Bajaj , R. M. Soriano , and C. Shermetaro , Anatomy, Head and Neck, Temporoparietal Fascia (Treasure Island (FL): StatPearls Publishing, 2024). 29939689

[10] C. DeLorenzi , “Complications of Injectable Fillers, Part 2: Vascular Complications,” Aesthetic Surgery Journal 34, no. 4 (2014): 584–600. 24692598 10.1177/1090820X14525035

[11] R. Ren , H. Xue , Z. Gao , et al., “Restoring Long‐Lasting Midface Volume in the Asian Face With a Hyaluronic Acid Filler: A Randomized Controlled Multicenter Study,” Journal of Cosmetic Dermatology 23, no. 6 (2024): 1985–1991. 38487954 10.1111/jocd.16221

[12] S. Oh , S. B. Seo , G. Kim , S. Batsukh , K. H. Son , and K. Byun , “Poly‐D,L‐Lactic Acid Stimulates Angiogenesis and Collagen Synthesis in Aged Animal Skin,” International Journal of Molecular Sciences 24, no. 9 (2023): 7986, 10.3390/ijms24097986. 37175693 PMC10178436

[13] S. B. Seo , H. Park , J. Y. Jo , and H. J. Ryu , “Skin Rejuvenation Effect of the Combined PDLLA and Non Cross‐Linked Hyaluronic Acid: A Preliminary Study,” Journal of Cosmetic Dermatology 23, no. 3 (2024): 794–802. 37969055 10.1111/jocd.16085

[14] S. Y. Chen , S. T. Chen , J. Y. Lin , and C. Y. Lin , “Reconstitution of Injectable Poly‐d,l‐Lactic Acid: Efficacy of Different Diluents and a New Accelerating Method,” Plastic and Reconstructive Surgery. Global Open 8, no. 5 (2020): e2829. 33154871 10.1097/GOX.0000000000002829 PMC7605845

[15] K.‐A. Byun , S. B. Seo , S. Oh , J.‐W. Jang , K. H. Son , and K. Byun , “Poly‐D,L‐Lactic Acid Fillers Increase Subcutaneous Adipose Tissue Volume by Promoting Adipogenesis in Aged Animal Skin,” International Journal of Molecular Sciences 25, no. 23 (2024): 12739. 39684448 10.3390/ijms252312739 PMC11641794

